# SHORT PHYSICAL PERFORMANCE BATTERY OUTPERFORMS COMMON PHYSICAL PERFORMANCE TESTS IN OLDER ADULTS

**DOI:** 10.1093/geroni/igae098.2614

**Published:** 2024-12-31

**Authors:** Chitra Banarjee, Renoa Choudhury, Joon-Hyuk Park, Rui Xie, David Fukuda, Jeffrey Stout, Ladda Thiamwong

**Affiliations:** University of Central Florida, Orlando, Florida, United States; University of Central Florida, Orlando, Florida, United States; University of Central Florida, Orlando, Florida, United States; University of Central Florida, Orlando, Florida, United States; University of Central Florida, Orlando, Florida, United States; University of Central Florida, Orlando, Florida, United States; University of Central Florida, Orlando, Florida, United States

## Abstract

Interdisciplinary evaluation of older adults’ healthcare is a priority in the prevention of chronic health conditions and the maintenance of daily functioning. While studies have evaluated different physical performance tests (PPTs) from a retrospective view in predicting mortality or cardiopulmonary health, it remains unclear which of the commonly used PPTs are most effective at evaluating the current health of older adults. Various PPTs have been shown to be important predictors of mortality; however, few studies have compared the relevance of different PPTs to older adults’ physical activity, static balance, body mass index, and fear of falling within the same population of older adults. The present study evaluates 53 older adults using three different PPTs Short Physical Performance Battery (SPPB), 6-minute walk test (6MWT), and Incremental Shuttle Walk Test (ISWT)) and constructs multiple linear regression models with measures of physical activity, static balance, and fear of falling, as assessed by an accelerometer, BTrackS balance plate, and the short Falls Efficacy Scale-International (FES-I), respectively. The models reveal that SPPB provides the most comprehensive value as indicated by a greater R2 value (0.573), and performance on the SPPB predicts physical activity (p=0.041) and fear of falling (p< 0.001). ISWT predicted body mass index (BMI, p=0.019) and fear of falling (0.010), whereas 6MWT predicted only fear of falling. The results indicate the value of a multi-component, comprehensive test such as the SPPB in evaluating the health of older adults and further distinguish ISWT as more predictive of overall health than the 6MWT in older adults.

